# Magnetic Resonance Elastography of Liver in Light Chain Amyloidosis

**DOI:** 10.3390/jcm8050739

**Published:** 2019-05-23

**Authors:** Sudhakar K. Venkatesh, Safa Hoodeshenas, Sandeep H. Venkatesh, Angela Dispenzieri, Morie A. Gertz, Michael S. Torbenson, Richard L. Ehman

**Affiliations:** 1 Department of Radiology, Mayo Clinic College of Medicine, Mayo Clinic, Rochester, MN 55905, USA; hoodeshenas.safa@mayo.edu (S.H.); ehman.richard@mayo.edu (R.L.E.); 2 Department of Radiology, Sengkang General Hospital, 110 Sengkang East Way, Singapore 544886, Singapore; Sandeep.halagatti.venkatesh@singhealth.com.sg; 3 Department of Medicine, Mayo Clinic College of Medicine, Mayo Clinic, Rochester, MN 55905, USA; dispenzieri.angela@mayo.edu (A.D.); gertz.morie@mayo.edu (M.A.G.); 4 Department of Laboratory Medicine and Pathology, Mayo Clinic College of Medicine, Mayo Clinic, Rochester, MN 55905, USA; torbenson.michael@mayo.edu

**Keywords:** hepatic amyloidosis, magnetic resonance elastography, liver stiffness, liver span, serum alkaline phosphatase

## Abstract

In this paper, we present our preliminary findings regarding magnetic resonance elastography (MRE) on the livers of 10 patients with systemic amyloidosis. Mean liver stiffness measurements (LSM) and spleen stiffness measurements (SSM) were obtained. Magnetic resonance imaging (MRI) images were analyzed for the distribution pattern of amyloid deposition. Pearson correlation analysis was performed in order to study the correlation between LSM, SSM, liver span, liver volume, spleen span, spleen volume, serum alkaline phosphatase (ALP), N-terminal pro b-type natriuretic peptide (NT pro BNP), and the kappa and lambda free light chains. An increase in mean LSM was seen in all patients. Pearson correlation analysis showed a statistically significant correlation between LSM and liver volume (*r* = 0.78, *p* = 0.007) and kappa chain level (*r* = 0.65, *p* = 0.04). Interestingly, LSM did not correlate significantly with SSM (*r* = 0.45, *p* = 0.18), liver span (*r* = 0.57, *p* = 0.08), or serum ALP (*r* = 0.60, *p* = 0.07). However, LSM correlated significantly with serum ALP when corrected for liver volume (partial correlation, *r* = 0.71, *p* = 0.03) and NT pro BNP levels (partial correlation, *r* = 0.68, *p* = 0.04). MRI review revealed that amyloid deposition in the liver can be diffuse, lobar, or focal. MRE is useful for the evaluation of hepatic amyloidosis and shows increased stiffness in hepatic amyloidosis. MRE has the potential to be a non-invasive quantitative imaging marker for hepatic amyloidosis.

## 1. Introduction

Hepatic involvement in systemic amyloidosis is common. It is found in 22%–95% of patients with a higher prevalence of primary amyloidosis [1,2,3,4,5]. Hepatic amyloidosis (HA) is characterized by the deposition of amyloid fibrils in the space of Disse that usually begins in the periportal region, although occasionally it is centrilobular and may also be deposited in the hepatic vasculature [4,6]. In severe cases, amyloid deposition leads to pressure atrophy of the hepatocytes, which interferes with the passage of bile resulting in cholestasis, or it may block the sinusoids, leading to portal hypertension [4,6,7,8]. HA is usually clinically silent, and hepatomegaly and mildly elevated serum alkaline phosphatase are the most common clinical manifestations [1,2,3,4,9,10,11]. Severe cases may be associated with jaundice, portal hypertension, liver failure, and, rarely, the spontaneous rupturing of the liver [12]. Splenomegaly can be associated with HA in about 15%–30% of cases, occasionally leading to hyposplenism and an increased risk of serious infection [13,14]. Portal hypertension, a common manifestation of chronic liver disease, and cirrhosis are fortunately rare in hepatic amyloidosis. Hepatic failure is also rare, except in advanced diseases, and hyperbilirubinemia, when present, is associated with poor prognosis [11]. Although HA rarely presents with clinical manifestations, it is associated with poor prognosis and the reduced survival of untreated patients [15,16]. Early diagnosis is the key to effective therapy, and treatment methods include chemotherapy, autologous stem cell transplantation, and liver transplantation. With the increased availability of novel chemotherapy, survival has improved significantly. However, there are no accurate quantitative measures available for assessing treatment response. Currently, liver involvement and response assessment is performed by measuring liver span on imaging modalities and correlating this with serum alkaline phosphatase levels. However, these measures are not very reliable. The degree of hepatomegaly and liver function abnormalities do not correlate with the amount of amyloid deposited in the liver [3,9]. Liver biopsy in these patients is controversial, as significant bleeding can occur owing to vascular involvement by amyloidosis, which may not constrict or clot normally, as well as the reduced hepatic synthesis of clotting factors and the relative deficiency of factor X due to binding to amyloid fibrils and clearance from circulation [3,5,17,18,19].

There is an unmet clinical need for the non-invasive evaluation of HA. Radiological findings of HA include non-specific-hepatomegaly, increased echogenicity on ultrasound or density with computed tomography (CT), and an increase in T1 signal intensity with magnetic resonance imaging (MRI) [20,21]. Scintigraphy with Tc-99m bound tracers shows heterogeneous uptake but is non-specific [22,23]. HA has been shown to increase liver stiffness measured with elastography [24,25,26,27,28]; however, there are few single case reports. Magnetic resonance elastography (MRE) is currently the most accurate non-invasive technique to detect and stage liver fibrosis [29,30]. MRE is useful for the detection of progression, treatment response, and the prediction of hepatic decompensation in patients with liver fibrosis [31,32]. MRE may be useful in the evaluation of HA given its proven value in hepatic fibrosis, as amyloidosis is also an extracellular process and associated with increased stiffness. Experience with MRE in HA is limited to a few single case reports [28]. In this study, we retrospectively evaluated 10 patients with HA who had undergone liver MRE, and we correlated liver stiffness measurement (LSM) with current methods of assessment of HA.

## 2. Methods

In this institutional review board-approved retrospective study, we searched for patients with amyloidosis who had also undergone a liver MRE study. Twelve patients with amyloidosis who had undergone liver MRE during a four-year period between 2014 and 2018 were found. Two of these patients were excluded from the study. One had undergone a splenectomy because of a possible rupture and had received treatment with Revlimid for 13 years, which resulted in the complete remission of amyloidosis. The other patient had rheumatoid arthritis and had received methotrexate. Methotrexate is known to cause liver fibrosis, a potential confounding factor for LSM. The final study group consisted of 10 patients.

Laboratory test results performed within 3 weeks (mean 4 days, range: 0–21 days) of liver MRE were obtained. The following laboratory tests were used in this study: serum alkaline phosphatase (ALP) (normal, 45–115 U/L), N-terminal pro b-type natriuretic peptide (NT pro BNP) (normal, ≤88 pg/mL), kappa (κ) free light chain (normal, 0.33–1.94 mg/dL), and lambda (λ) free light chain (normal, 0.55–2.63 mg/dL). HA was confirmed with biopsies obtained from the liver (5 cases). In the remaining 5 patients, HA was confirmed using the defined criteria of hepatomegaly (total liver span > 15 cm by imaging) or serum ALP ≥ 1.5 times the upper limit of normal and the confirmation of amyloidosis from biopsies from one or more other organs. In these 5 patients, systemic amyloidosis was confirmed with biopsies from bone marrow (4 cases), kidneys (2 cases), abdominal fat (2 cases), and lungs (1 case) [33]. The diagnosis of immunoglobulin light chain (AL) amyloidosis was established with mass spectrometry, which is routinely performed in our institute on all patients with suspected amyloidosis.

MRE of the liver was performed with a standard clinical liver MRE sequence, which is a two-dimensional gradient recalled echo (2DGRE) MRE as described previously [34]. Four slices of 10 mm thickness were obtained from the largest cross-section of the liver. The sequence produced magnitude and phase images immediately after acquisition. An inversion algorithm installed in the scanner processed the magnitude and phase images and produced stiffness maps automatically, which were available for review within a few minutes of acquisition of the MRE sequence. Mean liver stiffness measurement and mean spleen stiffness measurement (SSM) were measured by drawing large regions of interest over the liver and spleen on the stiffness maps using magnitude (anatomical) images as a reference The stiffness was recorded as kilopascals (kPa). For the evaluation of liver fibrosis, a color stiffness map with a standard scale of 0–8 kPa was produced along with a 95% confidence map overlay. A color map of 0–20 kPa was also produced. These are rarely used for clinical evaluation of liver fibrosis but may be useful in HA. The mean LSM and mean SSM were recorded. Two readers independently performed the LSM and SSM measurements on each case in order to assess reproducibility.

The maximum craniocaudal length of the liver (liver span) and spleen (spleen span) were measured on coronal T2-weighted images, which are routinely obtained for liver MRI study. The liver span is not an accurate measurement of hepatomegaly, as different lobes may be affected and there may be a combination of atrophy and hypertrophy. Therefore, we performed volumetry of the liver and spleen as they provide a more accurate assessment of liver and spleen enlargement. The liver volume and spleen volume were measured on a three-dimensional T1-weighted sequence by using the three-dimensional (3D) volumetry software available on our picture archival and communication server (PACS) (Visage Imaging, Inc., San Diego, CA, USA). All the measurements were performed by one reader.

Statistical analysis was performed using MedCalc Statistical Software v6.4.3 (MedCalc Software bvba, Ostend, Belgium; https://www.medcalc.org; 2016). The interobserver reliability and agreement analysis was performed with intra-class correlation analysis (ICC) and generated Bland–Altman plots with 95% agreement limits. Pearson correlation analysis was performed for correlation between continuous variables: LSM, SSM, liver span, spleen span, liver volume, spleen volume, serum ALP, NT pro BNP, κ chain and λ chain levels. Partial correlation analysis for the current methods for evaluation of HA liver span and serum ALP with LSM was performed. Partial correlation coefficient analysis is performed when one suspects that the relationship between two variables is influenced by other variables (covariates). The partial correlation coefficient can be adjusted or corrected for the influence of different covariates. Statistical significance was present when the *p*-value was ≤0.05.

## 3. Results

The final study group of 10 patients was composed of six men and four women with a mean ± SD age of 56.8 ± 9 years (range: 42–68 years). Hepatomegaly was found in eight patients. Ultrasound confirmed hepatomegaly in one patient, and in one patient, multiple hypodense lesions were found in the liver on a follow-up CT. Indications for MRI and MRE were as follows: follow-up imaging (4 patients), evaluated hepatomegaly (4 patients), raised total bilirubin (1 patient), and evaluation of focal lesions (1 patient).

**Figure 1:**
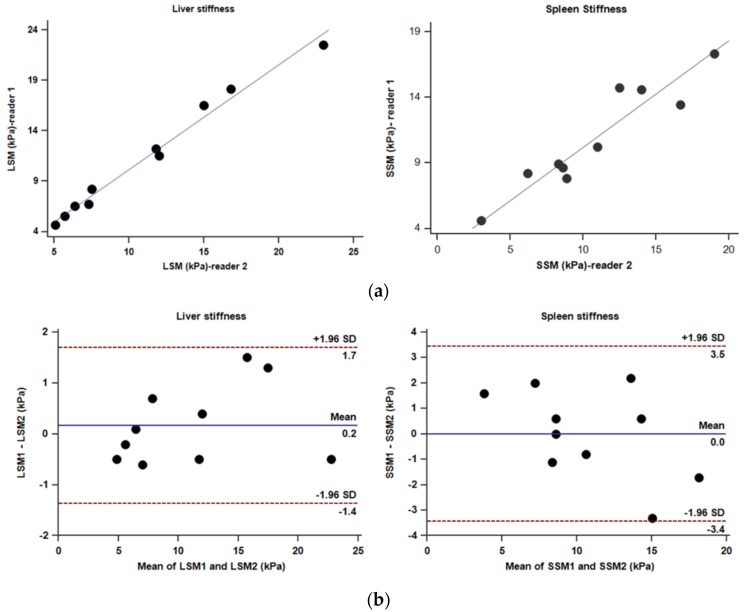
(**a**) Scatter plots showing excellent correlation between two independent readers for liver stiffness (*r* = 0.99) and spleen stiffness (*r* = 0.94). (**b**) Bland–Altman plots for liver stiffness and spleen stiffness. (LSM = liver stiffness measurement; SSM = spleen stiffness measurement)

The mean ± SD values of laboratory tests, liver span, liver volume, spleen span, spleen volume, LSM, and SSM are summarized in Table 1. Liver MRE studies were successful in all 10 patients. The mean LSM of the study group was 11.15 kPa (range: 4.85–22.75 kPa), which is at least 2 times the normal LSM (<2.5 kPa) [29,30,34]. The intra-class coefficient between two readers was excellent for both LSM (ICC 0.99, 95% CI, 0.98–0.99) and SSM (ICC 0.96, 95% CI, 0.85–0.99). The coefficient of repeatability with Bland–Altman plot analysis was 1.49 (95% CI, 1.08–2.61) for LSM and 3.27 (95% CI, 2.28–5.73) for SSM (Figure 1).

Pearson correlation analysis showed statistically significant correlation between LSM and liver volume (*r* = 0.78, *p* = 0.007), kappa chain level (*r* = 0.65, *p* = 0.04), and kappa/lambda ratio (*r* = 0.70, *p* = 0.02) (Table 2). Interestingly, LSM did not correlate significantly with SSM (*r* = 0.45, *p* = 0.18), liver span (*r* = 0.57, *p* = 0.08), or serum ALP (*r* = 0.60, *p* = 0.07). However, LSM correlated significantly with serum ALP when corrected for liver span (partial correlation coefficient, *r* = 0.71, *p* = 0.03) and NT pro BNP levels (partial correlation coefficient, *r* = 0.68, *p* = 0.04). The liver span did not show any significant correlation with LSM, even after correcting for liver volume or serum ALP. SSM correlated significantly with spleen volume (*r* = 0.83, *p* = 0.003).

**Figure 2:**
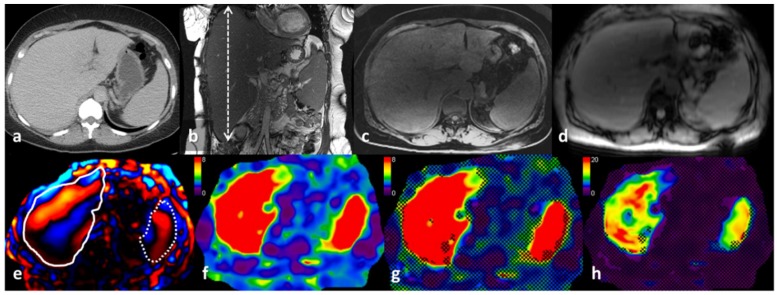
A 49-year-old female with systemic amyloidosis and hepatosplenomegaly on follow up. The axial computed tomography (CT) image (**a**), coronal T2-weighted image (**b**), and axial T1-weighted image (**c**) show hepatomegaly (craniocaudal span in (**b)**, 30 cm) and splenomegaly (16 cm). Axial magnitude resonance elastography (MRE) image (**d**), color wave image (**e**), and color stiffness maps (**f**–**h**) in the upper abdomen. The liver is outlined with a continuous line, and the spleen is outlined with a dotted line on the color wave image (**e**). The color stiffness map (**f**) shows stiffness distribution on the routinely used scale of 0–8 kPa. Note that the entire liver shows an increase in stiffness. The color stiffness map (**g**) is the same, with a 95% confidence overlay. The regions that are not crossed out are regions with reliable measurements. Note that both the liver and spleen are almost completely outlined by the overlay. The color stiffness map (**h**) has a wider scale of 0–20 kPa, and one can see the heterogeneity in the distribution of stiffness. This scale is rarely used in the clinical evaluation of liver fibrosis. The mean ± SD for LSM is 18.1 ± 3.4 kPa, and for SSM, it is 16 ± 2.1 kPa.

**Figure 3:**
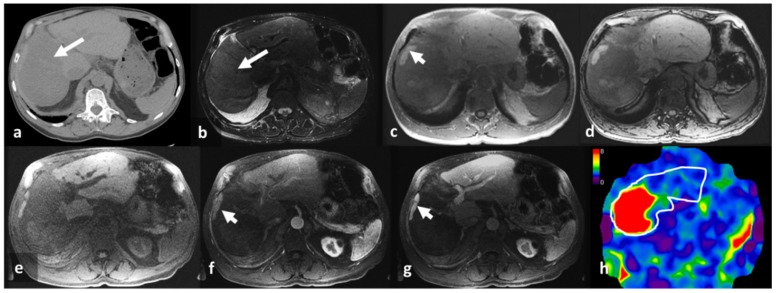
A 68-year-old male with known systemic amyloidosis and post autologous stem cell transplant on follow up. The axial non-contrast enhanced CT image (**a**) shows hypodense in the right lobe (arrow). The axial T2-weighted image (**b**) shows hyperintensity in the right lobe corresponding to the hypodensity in that lobe. In-phase (**c**) and opposed-phase (**d**) images show no significant signal loss (<5%) in the right lobe, confirming that there is no increase in fat. Note the small subcapsular area of sparing in the right lobe (arrowhead, (**c**)). The non-contrast enhanced (**e**), arterial phase (**f**), and portal venous phase (**g**) images show no significant enhancement in the right lobe but do show normal enhancement in the left lobe. The subcapsular focal area in the right lobe (arrow heads) shows similar enhancement to the left lobe, suggesting a spared region of normal liver parenchyma. The MRE stiffness map with a scale of 0–8 kPa (**h**) shows increased stiffness in the right lobe, corresponding to the abnormality seen on CT and magnetic resonance imaging (MRI), and normal stiffness in the left lobe. The mean ± SD LSM is 6.7 ± 1.2 kPa.

**Figure 4:**
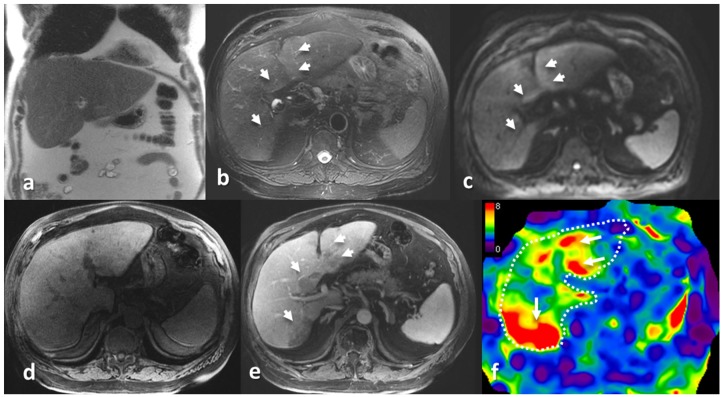
A 64-year-old male with known pulmonary amyloidosis on follow up. A follow-up CT (not shown) revealed hypodense lesions in the liver. An MRI with MRE was performed. The coronal T2-weighted image (**a**) shows borderline hepatomegaly (15.5 cm). The spleen was also borderline enlarged (12 cm). Multifocal lesions (arrow heads) were found in both lobes of the liver, which appeared hyperintense on the axial T2-weighted image (**b**) and diffusion weighted image (**c**), hypointense on the T1-weighted image (**d**), and hypoenhancing on the post contrast image (**e**). The MRE stiffness map (**f**) with a scale of 0–8 kPa shows moderately elevated liver stiffness (mean ± SD, 4.6 ± 0.8 kPa) with focal increased stiffness regions corresponding to the focal lesions. A percutaneous biopsy performed on the right lobe lesions confirmed hepatic amyloidosis.

A qualitative review of the stiffness maps with MRI images show that stiffness distribution was variable. The stiffer regions on the stiffness maps correspond to amyloid deposition. The most common presentation was a diffuse increase in stiffness (Figure 2). However, there was also single lobe involvement (Figure 3) and multiple focal lesions without significant hepatomegaly (Figure 4).

## 4. Discussion

Our preliminary study shows that LSM in light chain amyloidosis is higher than in normal liver stiffness and confirms the findings from previous case reports and studies with elastography [24,25,26,27,28,32]. LSM with MRE has an excellent interobserver agreement. LSM significantly correlated with liver volume but did not reach statistically significant correlation with liver span and serum ALP levels, which are the current methods for the evaluation of hepatic involvement in amyloidosis. However, there was a significant correlation with serum ALP when corrected for liver size and NT pro BNP levels. This may be due to the small number of patients in our study, and we also had cases with focal and heterogeneous involvement of the liver, which may have affected the serum ALP levels. LSM showed excellent correlation with liver volume, which can provide an accurate measurement of liver size. Amyloid deposition can result in non-uniform involvement of the lobes of the liver, and traditional right lobe measurement for hepatomegaly may not be applicable in such cases. HA leads to a heterogeneous and severe increase in liver stiffness compared to liver fibrosis or stiffness. Reviewing the wider scale stiffness map (0–20 kPa) is useful to appreciate this feature in addition to LSM.

We observed a trend of correlation between LSM and κ chain levels and no significant correlation with λ chain levels. This may because the κ chain was predominant AL type (6 cases versus 4 cases) in our series. Our results are consistent with the recently published series which showed that k chain deposition was more likely to be associated with AL hepatic amyloidosis. However our series is small and future studies with larger number of patients are needed to confirm this correlation [35].

Passive congestion can lead to increased LSM [36,37,38]. Cardiac involvement is frequent in amyloidosis and can cause cardiac failure. Passive congestion can, therefore, occur in these cases and contribute to the LSM. In our study, LSM did not significantly correlate with NT pro BNP levels; however, our study was small, and future studies comparing patients with and without congestive cardiac failure would be useful in establishing the contribution of passive congestion to LSM in HA.

In three of our cases, we observed the heterogeneous distribution of increased stiffness and, in one case, focal lesions in the liver. With a liver biopsy, these focal lesions were confirmed to be amyloid deposition. While it is known that HA is usually a diffuse process, heterogeneous or focal deposition can occur, and in these patients, there may be no hepatomegaly. MRI with MRE may be useful to complement clinical evaluation of hepatomegaly and serum ALP levels.

SSM did not significantly correlate with LSM, although it did correlate with spleen volume. This finding is different from previous experiences with liver fibrosis, where LSM correlated with SSM, which was thought to be due to increased portal pressure and passive congestion of the spleen [39]. However, the spleen may be involved in amyloidosis, which would also increase SSM regardless of portal pressure. Further portal hypertension is rare in hepatic amyloidosis, suggesting that amyloid deposition usually does not cause significant sinusoidal obstruction. However, this needs to be confirmed in a larger study.

Our study has limitations. First, as it is a retrospective study and clinical indications for routine liver MRI in HA are uncommon, it involved only a small number of patients A prospective study including all patients with systemic amyloidosis would be helpful in determining the incidence of HA that may go undetected. Second, we did not perform liver biopsy confirmation in all patients. Liver biopsy is controversial due to the increased risk of bleeding. We, however, performed liver biopsy in four cases where amyloidosis was not suspected without any complications. Third, four of our patients had received stem cell therapy, which may have affected the LSM. However, we correlated with liver span and serum ALP within three weeks of the MRE, which provided correlation at the time of follow up. A recent study using transient elastography reported that a reduction in LSM from baseline values correlated with the therapeutic clearance of amyloid [40]. A prospective study with baseline and follow-up LSM with MRE would be useful in determining the role of MRE in the assessment of treatment response in HA.

In conclusion, our initial experience with a small number of HA patients has shown that LSM is higher than in normal liver stiffness and correlates with liver volume. Our preliminary observations also highlight the fact that amyloid deposition in the liver is heterogeneous, with focal, asymmetric, and diffuse deposition possible. Liver stiffness measurement is a potential marker for liver involvement in light chain amyloidosis.

## Figures and Tables

**Table 1:** Laboratory test results and liver and spleen size and stiffness measurements.

	Mean	SD	Minimum	Maximum
Age (years)	56.8	9.00	42	68
Serum ALP (39–100 U/L)	308.7	241.59	67	701
NT pro BNP (≤140 pg/mL)	387.8	471.87	37	1424
Kappa chain (0.33–1.94 mg/dL)	7.82	13.45	0.42	39.6
Lambda chain (0.55–2.63 mg/dL)	5.03	7.25	0.12	22
Liver span (cm)	20.57	4.56	15.50	30
Liver volume (mL)	2944.8	956.74	1708	4370
Liver stiffness measurement (kPa)	11.15	5.94	4.85	22.75
Spleen span (cm)	12.61	3.0	9.40	18.5
Spleen volume (mL)	462.7	274.5	130	1080
Spleen stiffness measurement (kPa)	10.83	4.34	3.8	18.15

ALP: alkaline phophatase; NT pro BNP: N-terminal pro b-type natriuretic peptide.

**Table 2:** Pearson correlation coefficients between multiple variables *.

	Age	Kappa	Lambda	Serum ALP	NT pro BNP	Liver Span	Liver Volume	LSM	Spleen Span	Spleen Volume	SSM
Age		0.35	−0.03	−0.36	0.31	−0.33	−0.08	−0.23	0.16	0.23	0.08
Kappa	0.35		−0.18	0.22	−0.23	0.23	0.63	0.65 (0.04)	0.45	0.686 (0.03)	0.44
Lambda	−0.03	−0.18		−0.28	−0.10	−0.27	−0.36	−0.26	−0.34	−0.05	0.03
Serum ALP	−0.36	0.22	−0.28		0.13	0.33	0.21	0.60	0.60	0.34	0.06
NT pro BNP	0.31	−0.23	−0.10	0.13		−0.30	−0.40	−0.32	−0.06	−0.24	−0.25
Liver span	−0.33	0.23	−0.27	0.33	−0.30		0.810 (0.004)	0.57	0.41	0.35	0.12
Liver volume	−0.08	0.63	−0.36	0.21	−0.40	0.810 (0.004)		0.78 (0.008)	0.36	0.41	0.19
LSM	−0.23	0.65 0.04	−0.26	0.60	−0.32	0.57	0.78 (0.008)		0.59	0.60	0.45
Spleen span	0.16	0.45	−0.34	0.60	−0.06	0.41	0.36	0.60		0.85 (0.002)	0.58
Spleen volume	0.23	0.69 0.03	−0.05	0.34	−0.24	0.35	0.41	0.60	0.85 (0.002)		0.83 (0.003)
SSM	0.08	0.44	0.03	0.06	−0.25	0.12	0.19	0.45	0.58	0.83 (0.003)	

* The numbers in parenthesis are *p*-values for Pearson correlation coefficients.
